# Phased uplift of the northeastern Tibetan Plateau inferred from a pollen record from Yinchuan Basin, northwestern China

**DOI:** 10.1038/s41598-017-16915-z

**Published:** 2017-12-21

**Authors:** Xinling Li, Qingzhen Hao, Mingjian Wei, Andrei A. Andreev, Junping Wang, Yanyan Tian, Xiaolei Li, Maotang Cai, Jianmin Hu, Wei Shi

**Affiliations:** 1 College of Resource, Environment and Tourism, The Capital Normal University, Beijing, 100048 China; 2 Key Laboratory of Cenozoic Geology and Environment, Institute of Geology and Geophysics, Chinese Academy of Sciences, PO Box 9825, Beijing, 100029 China; 3 College of Earth Science, University of Chinese Academy of Sciences, Beijing, 100049 China; 4 Institute of Geology and Mineralogy, University of Cologne, Zülpicher Str. 49a, Cologne, 50674 Germany; 5 Institute of Geology and Petroleum Technologies, Kazan Federal University, Kazan, 420008 Russia; 6 Institute of Geomechanics, Chinese Academy of Geological Sciences, Beijing, 100081 China

## Abstract

The uplift of the Tibetan Plateau (TP) significantly affected both regional and global climates. Although there is evidence that the Tibetan Plateau experienced uplift during the Quaternary, the timing and amplitude are poorly constrained. However, the increased availability of long sedimentary records of vegetation change provides an opportunity to reconstruct the timing of the uplift. Here, we present a well-dated, high-resolution pollen record for the last 2.6 Ma from the Yinchuan Basin, which was incised by the Yellow River with its source in the northeastern Tibetan Plateau. Variations in the *Artemisia*/Chenopodiaceae (A/C) ratio of the reveal changes in moisture conditions in the Yinchuan Basin during glacial-interglacial cycles, as well as a gradual long-term aridification trend which is consistent with progressive global cooling. However, fluctuations in the percentages of *Picea* and *Abies* differ from those of the A/C ratio and we propose that they reflect changes in the vegetation and environment of high elevation areas. The *Picea* and *Abies* records reveal two phases of increased representation, at 2.1 and 1.2 Ma, which may indicate phases in the uplift of the northeastern Tibetan Plateau. Thus, they provide independent evidence for the timing of the uplift of the Tibetan Plateau during the Quaternary.

## Introduction

The uplift of the Tibetan Plateau during the late Cenozoic had significant effects on both regional and global climate. They included the strengthening of monsoon systems, the possible initiation of Northern Hemisphere glaciation, the formation of a complex mosaic of vegetation comprising many regionally distinctive types, and the intensification of aridification in inner Asia^1–4^. The uplift may also have influenced global climate by accelerating rock weathering and withdrawing carbon dioxide from the atmosphere^5^.

The uplift history of the Tibetan Plateau during the Quaternary has been studied from various perspectives, including global and regional climate change^6,7^, tectonics^8^, oxygen isotopes^9,10^, aeolian deposition^11,12^, and erosion rates^13,14^. However, despite this intensive research effort the precise timing of the uplift of the Tibetan Plateau is still unclear and interpretations of the processes are controversial^15^.

Many quantitative paleoelevation methods can be used to measure changes in landscape elevation^16^. Among them, pollen records, which document changes in mountain vegetation, are especially useful for tracing the history of tectonic uplift^17,18^. Adjacent sedimentary basins receive abundant pollen influx from the surrounding mountains, especially through the inputs of fluvial detritus^19^. Coniferous trees, particularly spruce (*Picea*) and fir (*Abies*), are important components of mountain ecosystems and their pollen records are effective indicators of the elevation of the mountains. A study of surface pollen in the Tibetan Plateau and adjacent mountains in Xinjiang Autonomous Region showed that *Picea* and *Abies* pollen frequencies greater than 20% occurred in surface samples collected from between 2500 and 4000 m above sea level (a.s.l.)^20^. In addition, higher frequencies of *Picea* and *Abies* occurred in regions with a mean annual temperature between 0 and 8 °C and annual precipitation around 400–850 mm^21^. Moreover, frequencies of *Picea* and *Abies* of up to 20% occurred in surface samples from spruce and fir forests, while frequencies less than 5% were found in forests in which spruce and fir trees were absent, indicating that their representation in pollen spectra is closely related to the source plants^22^.

The representation of *Picea* and *Abies* in pollen diagrams has played an important role in paleo-elevation reconstruction. Both taxa are well represented in pollen diagrams from many regions of China and the expansion of spruce and fir are often interpreted as resulting from the uplift of the Tibetan Platea^23^. Pollen data from well-dated lacustrine sedimentary deposits in the Xining Basin show that the appearance and subsequent increase in *Picea* at ~36 Ma was coeval with the uplift of the Tibetan Plateau^24,25^. A 2.8 Ma pollen record from Co Ngoin Lake in the central Tibetan Plateau suggests that the appearance of mountainous dark coniferous forests was the result of tectonic activity^26^. In addition, high *Picea* percentages in a sediment record from the Lanzhou Basin were interpreted as a result of Tibetan Plateau uplift during the Oligocene^27^.

In the present study, we obtained a pollen record from sediment core PL02 drilled in the Yinchuan Basin on the northeastern margin of the Tibetan Plateau. Subsequently, we used changes in the pollen assemblages as an independent (although indirect) indicator to identify phases in the uplift of the Tibetan Plateau.

**Figure 1:**
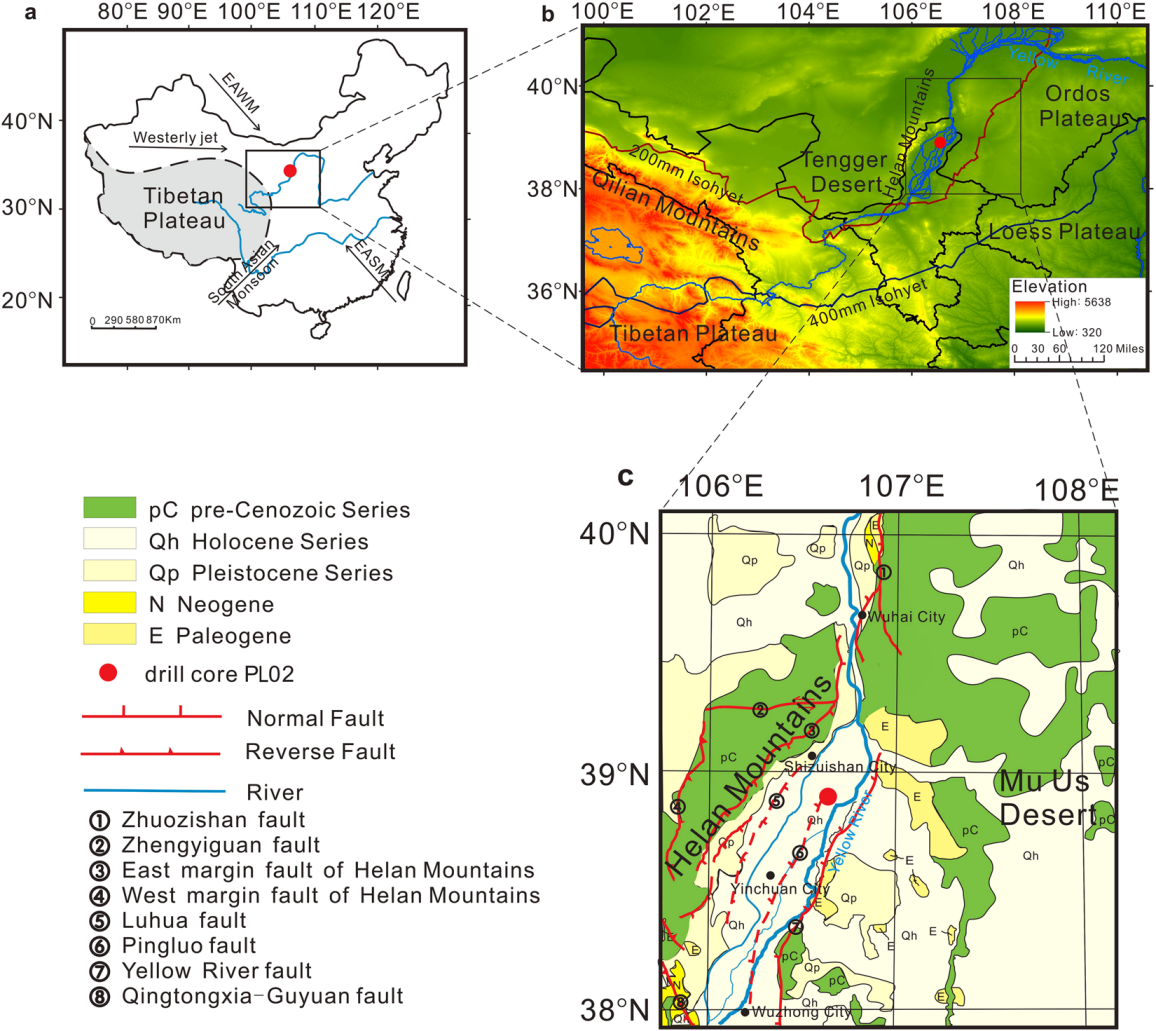
Environmental setting of the study area. (**a**) Map of China showing the major atmospheric circulation systems: EAWM - East Asian Winter Monsoon and EASM - East Asian Summer Monsoon. (**b**) Digital elevation map of northern China showing the northeastern Tibetan Plateau, the Yellow River and the Yinchuan Plain. The map was generated using ArcGIS 10.0 (https://www.arcgis.com/features/), elevation data were obtained from the Digital Elevation Model (DEM) (90 m × 90 m), provided by the International Scientific & Technical Data Mirror Site, Computer Network Information Center, Chinese Academy of Sciences (http://www.gscloud.cn). (**c**) Geological map showing the location of drill core PL02. Figure 1a and c were all produced using CorelDraw Graphics Suite 2017 (http://www.coreldraw.com/cn/?topNav=cn).

Yinchuan Basin is a graben basin situated between the Helan Mountains and the Ordos Plateau, northeast of the Tibetan Plateau and northwest of the Loess Plateau (Fig. 1a,b). The Yellow River, which originates in the Tibetan Plateau, runs through the basin from south to north and carries large amounts of sediment into the basin. Sedimentation in the basin has occurred during much of the Cenozoic, with the thickness of Quaternary sediments reaching some 2000 m^28^, and these sediments are a major archive of paleoenvironmental information. The Yinchuan Basin is in the transition zone between the region to the east with a warm and humid monsoon climate and the interior region with a cold and dry climate^29^. The basin is close to the modern 200 mm isohyet (Fig. 1b) which roughly delineates the border between arid and semi-arid climates in the region. Thus, the basin is well located for studying the long-term history of plateau uplift and changes in monsoon evolution.

The main natural vegetation type in the region is desert steppe (see Supplement Fig. 1). Desert steppe vegetation communities are dominated by xerophytes, including perennial grasses such as *Stipa breviflora*, *S. glareosa*, *S. tianschanica* var. *gobica*, *Ptilagrostis mongholica*, *P. dichotoma*, *Cleistogenes caespitosa*, and semishrubs such as *Oxytropis aciphylla*, *Reaumuria songarica*, and *Salsola passerina* ^30^. The vegetation of the Helan Mountains^31^ is represented by Qinghai spruce (*Picea crassifolia*), which is the most common tree occupying elevations between 2100 and 3100 m a. s. l. on shadowed and semi-shadowed slopes.

Core PL02 (38°55′26.62″N, 106°36′3.82″E, 1103 m a.s.l.) was drilled in the city of Shizuishan in Ningxia Hui Autonomous Region, where thick Quaternary sediments are preserved (Fig. 1b,c). The study area has a temperate arid climate with four distinct seasons: a warm and windy spring, a short hot summer, a short cool autumn, and a long cold winter^32^. The mean annual temperature for AD 1981–2010 was 10.1 °C, the annual precipitation was 167.1 mm, the mean temperature of the warmest month was 25.1 °C, and the mean temperature of the coldest month was −7 °C (see Supplement Fig. 2).

Core PL02 was obtained in September 2012. The total core length is 720.7 m with a recovery of 94.7%. The sedimentary record mainly comprises alluvial and fluvial deposits consisting of medium and fine sand and silt/clay, while the topmost 0.83 m consists of cultivated soil disturbed by human activity. Magnetostratigraphic analysis showed that the intervals of 0–137.2 m, 137.2–496.3 m, and 496.3–720 m correspond to the Brunhes, Matuyama and Gauss Сhrons, respectively^33^. The age of the base of the core basis is approximately 3.4 Ma.

The present study focuses on the pollen record of the upper 496.7 m, which documents environmental changes of the past 2.6 Ma (see Methods Summary). The chronology of the studied sequence is based on paleomagnetic dating, correlation of the ratio of the log transformed percentages of *Artemisia* and Chenopodiaceae pollen (*ln* A/C) with the marine oxygen isotope record (see Supplement Fig. 3), and loess stratigraphy (see Supplement Fig. 4).

A total of 324 samples were used for pollen analysis. Pollen samples were prepared using standard methods (see Methods Summary). At least of 150 terrestrial pollen grains (average of 206 grains) were counted for each sample. Pollen concentrations varied between 10 and 1115 grains/g (average 124 grains per gram).

## Results

**Figure 2:**
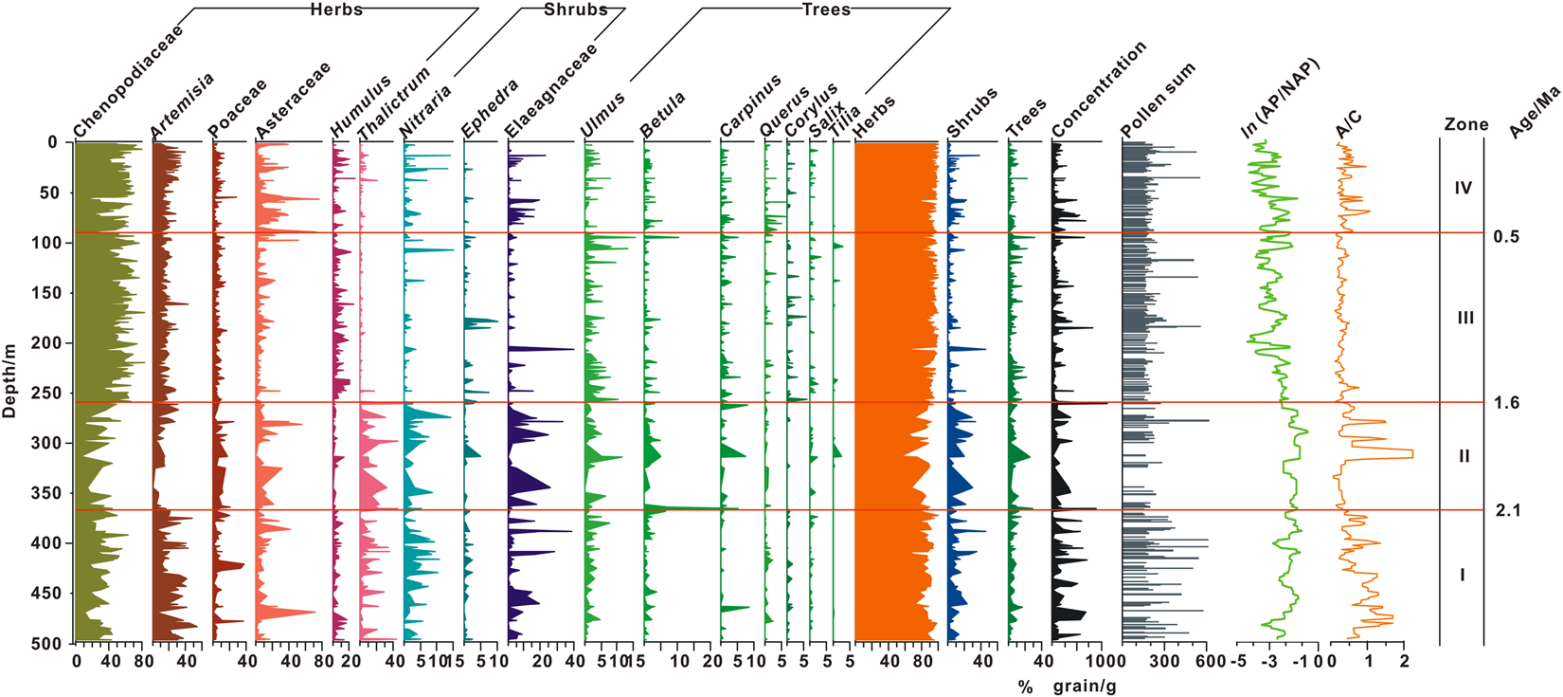
Pollen percentage diagram for core PL02 showing selected taxa. The pollen of *Pinus*, *Picea*, and *Abies* are likely of mountain origin and are supplied by long-distance transport. Thus, they were removed to highlight changes in local pollen components.

**Figure 3:**
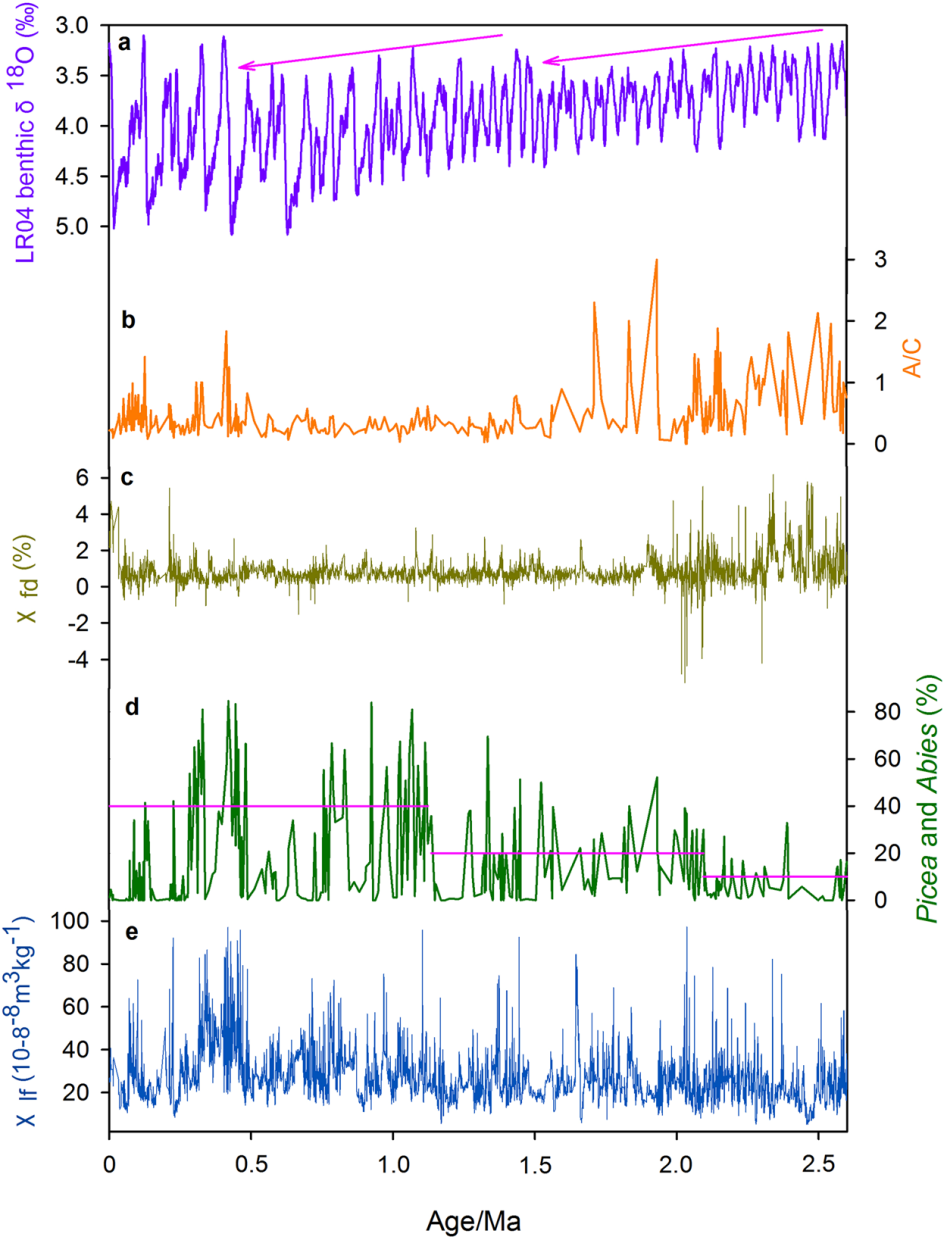
Comparison of records from core PL02 with the LR04 δ^18^O stack. (**a**) Benthic δ^18^O stack LR04^47^. (**b**) Ratio of the percentages of *Artemisia* and Chenopodiaceae (A/C). (**c**) Frequency-dependent magnetic susceptibility (χ_fd_). (**d**) Percentages of *Picea* and *Abies*. (**e**) Low-frequency mass magnetic susceptibility (χ_lf_).

A total of 69 pollen taxa were identified in the studied samples. The most common terrestrial taxa are the needleleaf trees *Pinus*, *Picea*, and *Abies*; the broadleaf trees *Quercus*, *Betula*, and *Ulmus*; the shrubs *Nitraria*, *Ephedra*, and Elaeagnaceae; and the herbs Poaceae, Chenopodiaceae, Asteraceae, *Artemisia*, and Rosaceae. The main aquatic taxon is *Typha*. The principal spores are Polypodiaceae and *Selaginella*. For calculation of individual taxa percentages, the sum of terrestrial taxa (broadleaf trees, herbs and shrubs) was used (Fig. 2). However, to highlight local pollen fluctuations, the pollen of *Pinus*, *Picea*, and *Abies*, which are mainly produced by mountain vegetation, was excluded (Fig. 2); the percentages of these taxa was based on the sum of needleleaf taxa and the remaining terrestrial taxa (Fig. 3).

The pollen spectra over the past 2.6 Ma are characterized by high percentages of herbs (average 90.0%) and shrubs (average 5.3%) and low percentages of trees (average 4.6%) (Fig. 2). Chenopodiaceae (average 46.0%) and *Artemisia* (average 17.1%) dominate the spectra. The ratio of *Artemisia* to Chenopodiaceae (A/C) exhibits a large amplitude of variation (from 0 to 3, average 0.5). The log-transformed ratio of arboreal pollen (AP) to non-arboreal pollen (NAP) ranges from −0.8 to −6.2 (average −3.3).

Cluster analysis was used to subdivide the pollen spectra into four major assemblage zones which are described below.

### *Zone I*(496.7–366 *m*, ∼2.6–2.1*Ma*)

Herbs (average 86.1%) and shrubs (average 9.2%) dominate the pollen spectra of this zone. The range and average of the main herb taxa are as follows: Chenopodiaceae - 11.2–63.7% (average 31.5%); *Artemisia* - 3.5–54.2% (average 22.2%); *Thalictrum* - 0–44% (average 8.5%), Asteraceae - 0–71.5% (average 8.2%), and Poaceae - 0.3–37.7% (average 6.9%). *Humulus* (average 3.9%), *Urtica* (average 1.9%) and Rosaceae (average 0.8%) are also represented. Shrub taxa include Elaeagnaceae (average 5.0%), *Nitraria* (average 2.9%), and *Ephedra* (average 0.6%). *Nitraria* is an arid-land shrub which is relatively well represented in the modern local vegetation in the Yinchuan Basin^31^. Modern surface pollen studies show that the frequency of *Nitraria* is a good indicator of local aridity^34,35^. In the study area, Elaeagnaceae mainly includes *Hippophae* and *Elaeagnus*. *Hippophae* is a mesic deciduous shrub^30^. *Elaeagnus* grows on riverbanks in desert and desert steppe areas^36^.

The application of principal components analysis to the pollen data from core PL02 revealed distinct groups of taxa which are probably related to variations in moisture conditions (see Supplement Fig. 5 and Methods Summary). *Nitraria* and Elaeagnaceae grow in an environment that is significantly more humid than that favored by *Artemisia* and Chenopodiaceae. This confirms that in arid and semiarid areas, the presence of shrub pollen indicates a slightly more humid environment than that reflected by the pollen of typical arid-land herbs.

Tree pollen percentages (mainly *Ulmus* and *Betula*) are low: *Ulmus* ranges from 0–7.5% (average 1.6%) and *Betula* from 0–6.3% (average 0.8%). Subtropical and temperate deciduous broadleaved trees include *Juglans*, *Eucommia*, and *Quercus* which are present in most samples of this zone I. The average percentage of broadleaf trees is 4.7%. The pollen concentration of the zone ranges from 10–701 grains/g (average 184 grains/g). The A/C ratio ranges from 0.1–2.1 (average 0.8), and the average value of the zone is the highest for the entire section. *ln* (AP/NAP) ratios range from −6.2 to −1.6 (average −3.3) (Fig. 2).

### *Zone II* (366–259 *m*, 2.1–1.6*Ma*)

This zone is characterized by the highest representation of trees (average 7.4%) throughout the entire sequence; shrubs (average 12.2%) are also well represented. The representation of herbs is lower (average 80.4%) compared to the previous zone. *Carpinus, Betula* and *Ulmus* have average values of 0.8%, 2.7% and − 2.0%, respectively. Other tree taxa, namely *Salix*, *Juglans*, *Tilia*, and *Eucommia*, are also represented but with values of less than 1%. The average representation of broadleaf trees is 7.4%.

The proportions of desert and steppe taxa, including Chenopodiaceae (average 28.0%) and *Artemisia* (average 11.2%), decrease remarkably compared to zone I; *Humulus* (average 1.9%) also decreases. In contrast, the representation of *Thalictrum* (mesic steppe taxa) increases significantly (average 13.8%), and that of the mesic Elaeagnaceae (ranging from 0 to 33.3%, average 7.9%) and Asteraceae (mostly mesic and arid taxa) (average 10.7%) also increase. The pollen concentration increases to a maximum of 1115 grains/g (average 191 grains/g). The A/C ratio decreases significantly (range from 0 to 3, average 0.6) compared to the previous zone; however, the *ln* (AP/NAP) ratio continues to maintain high values (range from −4.6 to −0.9, average −2.8) (Fig. 2).

### *Zone III* (259–89 *m*, 1.6–0.5*Ma*)

This zone is characterized by an abrupt increase in Chenopodiaceae (average 54.3%) which reaches a maximum within the entire sequence. *Humulus* (average 5.6%) also increases. The representation of other steppe and desert taxa including *Artemisia* (average 15.5%) also increases slightly compared to zone II, while the mesic *Thalictrum* decreases significantly (average 0.6%). Other mesic herbs and shrubs including Elaeagnaceae (average 1.3%) and Asteraceae (average 5.6%) also decrease. The representation of *Betula* and *Juglans* decreases slightly compared to zone II, while *Eucommia* and *Corylus* increase slightly. The representation of broadleaf trees decreases to an average of 5.3% (Fig. 2); however, other tree taxa (*Quercus*, *Ulmus*, and *Salix*) maintain a similar representation compared to the previous zone. The pollen concentration decreases (range of 11–820 grains/g, average 93 grains/g) and the A/C ratio decreases significantly (range of 0–0.8, average 0.3). The *ln* (AP/NAP) ratio decreases continuously within the zone (range from −5.3 to −0.8, average −3.2).

### *Zone IV* (89–0*m*, 2.5–0 *Ma*)

This zone is characterized by a significant increase in Asteraceae (average 12.6%). At the same time, Chenopodiaceae decreases (range of 9.2–80.2%, average 50.4%) and consequently the A/C ratio increases slightly (range of 0.1–1.8, average 0.4) compared to the previous zone. The representation of mesic and arid herbs and shrubs, including *Artemisia*, *Thalictrum*, and Elaeagnaceae, increase gradually, while that of the broadleaf trees (*Ulmus*, *Eucommia*, *Corylus*, *Carpinus*) decrease compared to zone III. The broadleaf trees percentage decreases to 3.1% on average (Fig. 2). The pollen concentration increases slightly (range of 12–684 grains/g, average 104 grains/g) and the *ln* (AP/NAP) ratio decreases slightly (range from −5.3 to −1.2, average −3.5).

## Discussion

Modern pollen-climate relationships provide a basis for the interpretation of changes in the pollen assemblages in core PL02. Studies of modern vegetation in arid and semi-arid areas indicate that the local vegetation depends mainly on the effective moisture. In the modern steppe and desert areas of north China, *Artemisia* and Chenopodiaceae frequently dominate plant communities^37^. *Artemisia* is an important component of steppe vegetation, while Chenopodiaceae is the most characteristic components of desert vegetation^38^. *Artemisia* requires greater humidity than Chenopodiaceae during the growing season^39–41^. The relative proportions of *Artemisia* and Chenopodiaceae in pollen assemblages are related to the degree of drought^42^. Various studies of arid and semi-arid regions show that A/C ratio is <0.5 in desert, 0.5–1.2 in desert steppe, and >1 in typical steppe^43–45^. Thus, the A/C ratio can be used to trace changes in the relative dominance of steppe and desert plants and thus variations in effective moisture in arid regions, with a lower ratio indicating drier conditions. Broadleaf trees grow in sunny and relatively warm regions and their high representation in pollen spectra thus reflects a relatively warm climate. Finally, the *ln* (AP/NAP) ratio is an indicator of the density of the tree cover^46^.

From the foregoing, we can conclude that the changes in the pollen assemblages in core PL02 reflect vegetation changes associated with the long-term aridification of the Yinchuan Basin since ~2.6 Ma, with the main environmental transitions occurring at ca. 2.1, 1.6, and 0.5 Ma (Fig. 2). *Artemisia*-Chenopodiaceae desert steppe was the dominant vegetation type in the basin between 2.6 and 2.1 Ma, and the average A/C ratios of ~0.8 suggest a desert steppe landscape during this period. The low *ln* (AP/NAP) values are also consistent with dry climatic conditions. Thus, we conclude that a desert steppe environment with a dry climate occurred in the study area during the early stage of the Quaternary (Fig. 2).

Between ca. 2.1 and 1.6 Ma, the environment of the basin was also dominated by desert steppe habitats; however, at the same time broadleaf trees such as *Ulmus*, *Betula* and *Carpinus* increased their representation within the vegetation. The percentages of shrubs and broadleaf trees are the highest within the entire sequence, indicating the warmest temperatures during the Quaternary. In addition, the total pollen concentration (reflecting vegetation productivity) and the *ln* (AP/NAP) ratios are also the highest within the sequence and the herb pollen content is the lowest. The denser vegetation coverage points to a wetter climate during the interval. Although on average the A/C ratio decreases within this interval, three peaks are evident in zone 2. The decrease in A/C ratio may be an artefact of the relatively low sampling resolution within this interval. In summary, we infer that relatively warm and moist conditions occurred in the study area between ca. 2.1 and 1.6 Ma (Fig. 2).

The increase in Chenopodiaceae and decrease in mesic herbs between 1.6 and 0.5 Ma suggest that the climate became extremely dry. The A/C ratio is the lowest (below 0.5) during the entire Quaternary, indicating the dominance of desert vegetation in the region (Fig. 2). The decrease in *ln* (AP/NAP) ratios from −2.8 to −3.2 indicates a decrease in tree cover, confirming the intensified aridity.

After 0.5 Ma, the representation of Chenopodiaceae decreased while *Artemisia* and the total pollen concentration increased. The A/C ratio (average 0.4) suggests the dominance of desert vegetation in the study area and overall the pollen assemblages point to the persistence of dry climatic conditions, although the humidity was greater than between 1.6 and 0.5 Ma (Fig. 2).

Overall the variations in the pollen stratigraphy of core PL02 correspond well to global and regional glacial-interglacial climatic changes, as shown by a comparison with benthic δ^18^O stack LR04^47^ and with paleoclimatic records from the Loess Plateau^48,49^ (Supplement Figs 3 and 4). However, because of differences in sampling resolution only the interval from 1.2 to 0 Ma (with a resolution of ~5 ka per sample) can be compared with the other records in terms of glacial and interglacial changes. As shown in Supplement Fig. 3, the peaks and troughs in the *ln* A/C curve compare well with the variations in LR04 for the last 1.2 Ma. For core PL02 the resolution is only ~10 ka per sample for the interval from 2.6 to 1.2 Ma, preventing a detailed comparison with the δ^18^O stack; however, a low resolution comparison can be made with records from the Loess Plateau (Supplement Fig. 4).

There is a close correspondence between the variations in the *ln* A/C record in core PL02 and benthic δ^18^O stack LR04 for the last 1.2 Ma, as well as with the Wucheng paleosol groups (WS) and the Wucheng loess units (WL) for the interval from 2.6–1.2 Ma interval (Supplement Figs 3 and 4). The comparison suggests that the sedimentary and paleoenvironmental record of core PL02 is continuous and coherent with global climate changes. The pollen record from core PL02 reflects gradual landscape changes from desert steppe to desert, and thus a trend of progressive aridification in the Yinchuan Basin during the Quaternary. Moreover, the fluctuations in aridity indicated by the *ln* A/C ratio are roughly synchronous with records of dust deposition during the Quaternary^50–52^. Thus, we conclude that the pollen data from core PL02 provide a continuous record of decreasing humidity in the arid region of northwestern China.

Core PL02 consists of alluvial-fluvial sediments and thus the pollen content is likely to be supplied by rivers and/or by winds. Consequently, the pollen record will reflect contributions from a large area, including from the adjacent mountain ranges. We assume that the pollen component of local origin, such as *Artemisia* and Chenopodiaceae, reliably portray a trend of aridification within the study area. In addition, on an orbital time scale, the fluctuations in *Picea* and *Abies* correlate well with the glacial-interglacial cycles observed in the marine δ^18^O record, with high (low) percentages in interglacials (glacials) (Fig. 3 and Supplement Fig. 3). However, these long-term changes in *Picea* and *Abies* differ significantly from those of taxa whose pollen is of local origin. The conifer pollen content exhibits a stepwise increase during interglacial phases of the last 2.6 Ma (Fig. 3); from their modern ecology, spruce and fir are representative of cold and wet climatic conditions^53^ and thus increases in their representation should reflect the development of these conditions. However, in contrast the variations in A/C ratio indicate a drying trend in the study area. At present, spruce grows at high elevations on the Tibetan Plateau and on other mountain ranges in China and its presence in pollen records has always been interpreted as a signal of the vegetation at high altitudes^54^. However, dry habitats are unsuitable for the growth of coniferous trees and since desert steppe is dominant in the Yinchuan Basin we can exclude the possibility that the pollen of *Picea* and *Abies* originated from the local desert steppe vegetation.

Pollen in arid and semi-arid regions is usually transported by wind and water^55,56^. A modern pollen study in the Shiyang River basin (eastern Qilian Mountains) showed that spruce pollen percentages fall to 1% and less when the distance from the source trees is more than 500 m, while rivers can transport pollen much greater distances^55^. The distance between the site of core PL02 and the Helan Mountains about 35 km and therefore the large amounts of *Picea* and *Abies* pollen in our record (up to 40–80%) cannot be transported from the Helan Mountains by wind. Moreover, spruce flowers from July to September when southeasterly winds are dominant in the study area, which significantly reduces the potential transport of *Picea* pollen from the Helan Mountains northwards towards the drilling location.

However, the Yellow River carries large volumes of suspended sediment which also contain large amounts of pollen from the adjacent mountains^56^. When the Yellow River flows from the plateau/canyon portion of the northeastern Tibetan Plateau into the Yinchuan Basin, its carrying decreases rapidly, in response to the decrease in channel gradient. Thus, the *Picea* and *Abies* pollen in core PL02 probably originated mainly from the northeastern part of the Tibetan Plateau and was transported by the Yellow River.

Two phases of increased *Picea* and *Abies* representation during interglacials are evident in the pollen record: at around 2.1 and 1.2 Ma (Fig. 3). There are four possible explanations for these increases: (i) increased precipitation in the northeastern Tibetan Plateau, (ii) global cooling, (iii) headward erosion of the Yellow River, and/or (iv) uplift of the Tibetan Plateau. We now examine each of these possibilities in turn.

### Increased precipitation

The climate of the northeastern Tibetan Plateau is similar to that of the arid Yinchuan Basin, where summer precipitation is dominated by the strengthened Asian summer monsoon. On a long-time scale, the climatic changes in the study are synchronous with those of the Loess Plateau. The increase in *Picea* and *Abies* percentages during interglacial periods (Fig. 3) can be attributed to increased precipitation. If this assumption were correct then the stepwise increases in *Picea* and *Abies* at around 2.1 and 1.2 Ma indicate significant increases in precipitation in the northeastern Tibetan Plateau. However, this inference disagrees with the trend of gradual aridification trend of both the Yinchuan Basin (Fig. 3b) and the Loess Plateau^52^. Therefore, we conclude that long-term changes in summer precipitation in Asia were not the main cause of the stepwise increases in *Picea* and *Abies* during interglacials.

### Global cooling

**Figure 4:**
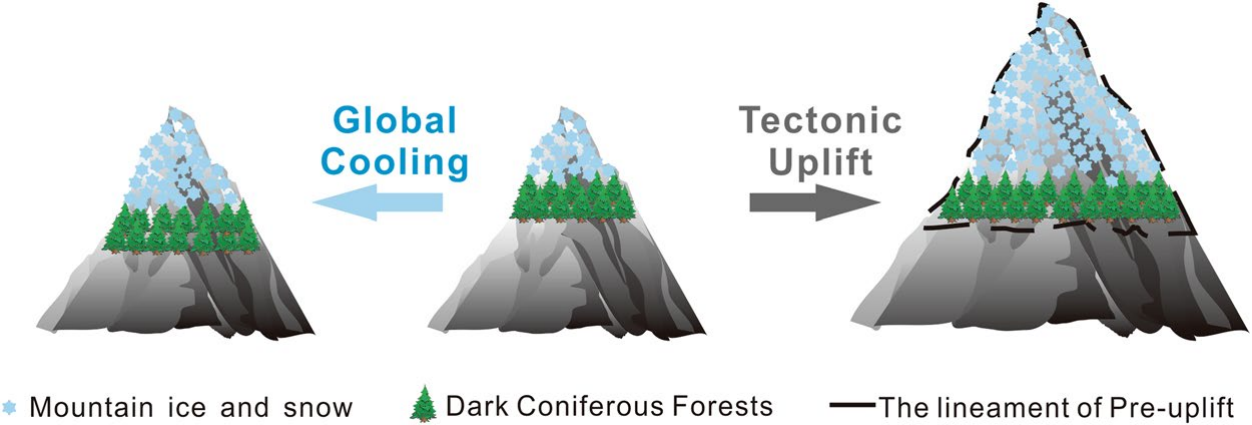
Schematic diagram illustrating changes in the altitudinal position of the dark coniferous forest zone under conditions of global cooling and tectonic uplift. The diagram was produced with CorelDraw Graphics Suite 2017 (http://www.coreldraw.com/cn/?topNav=cn).

The oxygen isotopic composition of sea water is controlled by global ice volume and sea-water temperature^47^. A recent reconstruction of global temperature over the past 2 million years parallels that of the marine δ^18^O record^57^, and thus changes in marine δ^18^O stacks are used to estimate the effect of global surface cooling during the past 2.6 Ma. Climatic cooling would lead to the downward displacement of the dark coniferous forest zone, resulting in an enlargement of the habitat of *Picea* and *Abies* (Fig. 4). As shown in Fig. 3a, there are two main stages of increase in interglacial temperature, at 1.6 and 0.6–0.4 Ma, which are superimposed on the long-term trend of decreasing global temperature. Thus, it is clear that the stepwise increases in *Picea* and *Abies* pollen during interglacial phases are inconsistent with the stepwise temperature changes (Fig. 3a and d) and therefore we conclude that global cooling was not the main cause of the pollen changes.

### Headward erosion

Headward erosion of the Yellow River may partly contribute to the increase representation of *Picea* and *Abies* since an increase in the erosion of high elevation portions of the drainage area may lead to the addition of sediments containing significant amounts of *Picea* and *Abies* pollen which are carried as part of the suspended load. The headward erosion is generally mainly controlled by two factors: climate change and surface uplift. The absence of a coupling of the stepwise changes in *Picea* and *Abies* with the global climate record (Fig. 3a and d), mentioned previously, precludes the possibility that increased headward erosion was mainly caused by climate change. Moreover, if headward erosion were a major cause of changes in the representation of *Picea* and *Abies*, then an increase in the erosion of the high elevation portions of the drainage area would have led to increases in their representation during both glacial and interglacial periods since the onset of the erosion. Thus, the overall low and uniform representation of *Picea* and *Abies* during glacial periods throughout the past 2.6 Ma (Fig. 3d) fails to support headward erosion as a cause. Nevertheless, the history of headward erosion of the Yellow River, and its possible influence on the pollen assemblages of river-transported materials, should be further investigated in the future.

### Uplift of the Tibetan Plateau

As we have demonstrated, the observed changes in *Picea* and *Abies* in core PL02 are difficult to explain by long-term changes in precipitation and temperature or by headward erosion of the Yellow River. Thus, we conclude that major stages in the uplift of the northeastern Tibetan Plateau at 2.1 and 1.2 Ma were likely responsible for the increased representation of *Picea* and *Abies*. As shown schematically in Fig. 4, in the absence of changes in other factors, tectonic uplift would result in an increase in the habitat area of the *Picea* and *Abies* forest belt and the pollen of these trees would be transported by the Yellow River and deposited in the Yinchuan Basin. Thus, we suggest that the increases in *Picea* and *Abies* at 2.1 and 1.2 Ma indicate two important uplift stages of the northeastern Tibetan Plateau.

During the last 2.6 Ma, there are several intervals with low frequencies of *Picea* and *Abies* within the interval of their overall increased representation: for example, from 0.75–0.49 Ma and from 0.28–0 Ma (Fig. 3). Although plateau uplift would lead to the expansion of the dark coniferous forest belts and the increased supply of conifer pollen to the Yinchuan Basin, these decreases cannot be explained by tectonic activity. Rather, these intervals of decreased *Picea* and *Abies* representation may be related to coeval arid episodes evident in the adjacent Loess Plateau. A continuous record of sand layers in the loess sequences along the loess-desert transitional zone shows that the Mu Us Desert migrated southward at 2.6, 1.2, 0.7 and 0.2 Ma^58^; however, the specific reasons for these episodes needs further study.

The pollen record from core PL02 from the Yinchuan Basin, in the climatically sensitive arid and semiarid transition zone, provides a continuous record of terrestrial environmental change during the Quaternary. Changes in the pollen spectra reflect the long-term aridification of the Yinchuan Basin since ~2.6 Ma, with major boundaries at ca. 2.1, 1.6, and 0.5 Ma. Stepwise increases in the representation of *Picea* and *Abies* during interglacial periods indicate that major phases in the uplift the Tibetan Plateau occurred at ca. 2.1 and 1.2 Ma.

## Methods Summary

### Pollen analysis

All samples were analyzed at Capital Normal University (Beijing, China). To ensure a sufficient pollen concentration for analysis, samples masses were adjusted according to lithology: 50 g for clay, 80 g for silt, 100 g for fine sand, 150 g for medium sand, and 250 g for coarse sand. A tablet containing a known number of *Lycopodium* spores was added to each sample to calculate the pollen concentration^59^. The samples were treated sequentially with 36% HCl to remove carbonate, with 40% HF to remove silica, and were then sieved through a 10 μm mesh to remove the fine fraction. The samples were then washed and mounted in glycerol. Pollen and spores were counted under an Olympus-BX51 light microscope at ×400 magnification in regularly spaced traverses for rough scanning, and at ×600 magnification for critical identifications. At least 150 pollen grains (average 206 grains) were counted for each sample. The total pollen concentration was calculated based on the sum of fossil pollen and *Lycopodium* spores in a sample and expressed as pollen grains per gram.

### Chronology

The chronology of core PL02 is based on magnetostratigraphy, which revealed 10 polarity reversals which defined a series of chrons and subchrons^33^. An initial time scale for the pollen data was established by linear interpolation between the polarity reversal boundaries which were used as first order tie-points. Then, the *ln* A/C ratio was correlated with the last 1.2 Ma interval of the benthic δ^18^O stack LR04^47^ (Supplement Fig. 3). New tie points were obtained for the time interval above the Jaramillo Subchron (Supplement Fig. 3). Three peaks in *ln* A/C ratio directly correspond with peaks in the LR04 record, eight secondary peaks resemble secondary peaks in LR04, and three troughs directly correspond with troughs in LR04. In addition, one secondary trough in *ln* A/C ratio corresponds to a trough in LR04, and a trough in *ln* A/C ratio corresponded to a secondary trough in LR04 (Supplement Fig. 3).

The fluctuations in *ln* A/C ratio were compared with the magnetic susceptibility record of the Lingtai section on the Chinese Loess Plateau^49^ between the base of the Jaramillo Subchron and the boundary of the Matuyama and Gauss Chrons (Supplement Fig. 4). The results of this comparison show that the PL02 pollen record below the Jaramillo matches well with the Wucheng paleosol groups (WS) and Wucheng loess units (WL)^48^ in the loess records. Because the ages of the top and bottom of the Olduvai Subchron in core PL02 are similar to those of the Lingtai section, the boundaries of the Olduvai Subchron were not tuned in our age model.

Based on the comparison of the *ln* A/C record in core PL02 and the LR04 δ^18^O stack (0–1.2 Ma interval) as well as with the Lingtai magnetic susceptibility record (1.2–2.6 Ma interval), 2^nd^ order tie-points were selected for core PL02. The final time scale was established by linear interpolation between tie points which included the paleomagnetic boundary ages and the 2^nd^ order tie points.

### Principal components analysis

Principal components analysis (PCA) was carried on selected pollen data from core PL02 (Supplement Fig. 5). The pollen concentrations of selected taxa with a minimum of 5 grains/100 g were analyzed. We interpret Axis 1 as representing a temperature gradient because of the positive correlation with *Ulmus* and negative correlation with *Picea* and *Abies*. *Ulmus* prefers sunlit slopes, suggesting that its increased representation reflects warmer conditions; in contrast, *Picea* and *Abies* generally occupy shadowed and semi-shadowed slopes, and thus their increased representation reflects cooler conditions. Axis 2 is interpreted as representing a humidity gradient, because of the relatively strong negative and positive correlations of Chenopodiaceae and Ranunculaceae, respectively. Chenopodiaceae species are the main components of the vegetation cover in desert steppe regions and they occupy dry habitats, while Ranunculaceae species prefer relatively humid environments. Thus, gradients in the PCA biplot mainly reflect differences between mountain coniferous (*Picea*-*Abies*) forests and semi-desert vegetation of the lower elevation plains. In addition, broadleaf taxa are positively correlated with both Axis 1 and Axis 2, indicating a preference of the broadleaf trees for a relatively warm and moist environment. The shrubs *Nitraria* and Elaeagnaceae are positively correlated with Axis 2, reflecting the fact that they prefer a slightly moister environment than herbs.

## Electronic supplementary material

Supplementary Information
