# Supplementary material for: Phased uplift of the northeastern Tibetan Plateau inferred from a pollen record from Yinchuan Basin, northwestern China
Source: Sci Rep. 2017 Dec 21;7:18023. doi: 10.1038/s41598-017-16915-z (PMC5740150; doi:10.1038/s41598-017-16915-z)
Supplement: Supplementary file 1 — Supplementary Information [file 41598_2017_16915_MOESM1_ESM.doc]

**Supplementary Information for**

**Phased uplift of the northeastern Tibetan Plateau inferred from a pollen record from Yinchuan Basin, northwestern China**

Xinling Li, Qingzhen Hao ^*^, Mingjian Wei ^*^, Andrei A. Andreev ^*^, Junping Wang, Yanyan Tian, Xiaolei Li, Maotang Cai, Jianmin Hu, Wei Shi

* Corresponding author. Prof. Qingzhen Hao, E-mail: [haoqz@mail.iggcas.ac.cn](mailto:haoqz@mail.iggcas.ac.cn); Prof. Mingjian Wei, E-mail: [weimj@mail.cnu.edu.cn](mailto:weimj@mail.cnu.edu.cn); Dr. Andrei A. Andreev, E-mail: [aandreev@uni-koeln.de](mailto:aandreev@uni-koeln.de).

**The modern vegetation of the Helan Mountains.** The mountain forest vegetation in the Helan Mountains^1^ is dominated by Qinghai spruce (*Picea crassifolia*) at elevations between 2100 and 3100 m on shadowed and semi-shadowed slopes^2^. The lower vegetation belt (1950-2200 m) is dominated by Chinese pine on shadowed and semi-shadowed slopes. On sunlit slopes, *Ulmus glaucescens* grows below 2700 m, and subalpine shrubs (*Potentilla glabra* and *P. parvifolia*) are dominant between 2700 and 3100 m. Slope differentiation of the vegetation is not obvious above 3000 m, where alpine shrubs (mostly *Salix cupularis* and *Caragana jubata*) dominate the vegetation, while alpine meadows with *Kobresia* only occur on the fertile thicker soils. A narrow steppe belt dominated by *Stipa capillata* occurs between 1800 and 1900 m. Mountain desert steppe of *Stipa glareosa*, *S. breviflora,* and *Artemisia frigida* covers the mountain slopes between 1600 and 1800 m, and steppe desert dominated by *Reaumuria soongarica* and *Salsola passerine* occurs below 1600 m.

**Determination of the inflections in the records of *Picea* and *Abies* at 2.1 and 1.2 Ma.** The determination of the inflections is based on the method of moving averages. As shown in Supplement Figure 6, the moving averages (3-, 5-, 8-, 10- and 20-point) of the percentages of *Picea* and *Abies* in core PL02 were calculated. With the increasing number of points in the calculation, any noise present in the record can be removed. The 10-point moving average exhibits a clear trend. The records of *Picea* and *Abies* exhibit three main phases during the Quaternary, with boundaries at ca. 2.1 and 1.2 Ma. In the 10-point moving average curve, the resolution is ca. 50 ka during the last 1.2 Ma and ca. 100 ka during 1.2-2.6 Ma. This resolution is appropriate for addressing the issue of tectonic-induced changes in the landscape.

**References**

1 Zhu, Z. Y., Liang, C. Z. & Li, Z. G., *Flora of Helan Mountain (in Chinese)* (ed. Zhu, F.) 0-848 (Sunshine press, 2011).

2 Ma, Y. Q., *Flora InnerMongolica (in Chinese)* 0-294 (Inner Mongolian People's Publishing House, 1985).

3 Lisiecki, L. E. & Raymo, M. E., A Pliocene-Pleistocene stack of 57 globally distributed benthic δ^18^O records. *Paleoceanography* **20**, PA1003, doi:10.1029/2004PA001071 (2005).

**Figure Legends**

**Figure S1. Vegetation maps of Yinchuan Basin.** The map was produced using CorelDRAW Graphics Suite 2017 ([http://www.coreldraw.com/cn/?topNav=cn](http://www.coreldraw.com/cn/free-trials/?topNav=cn)).

**
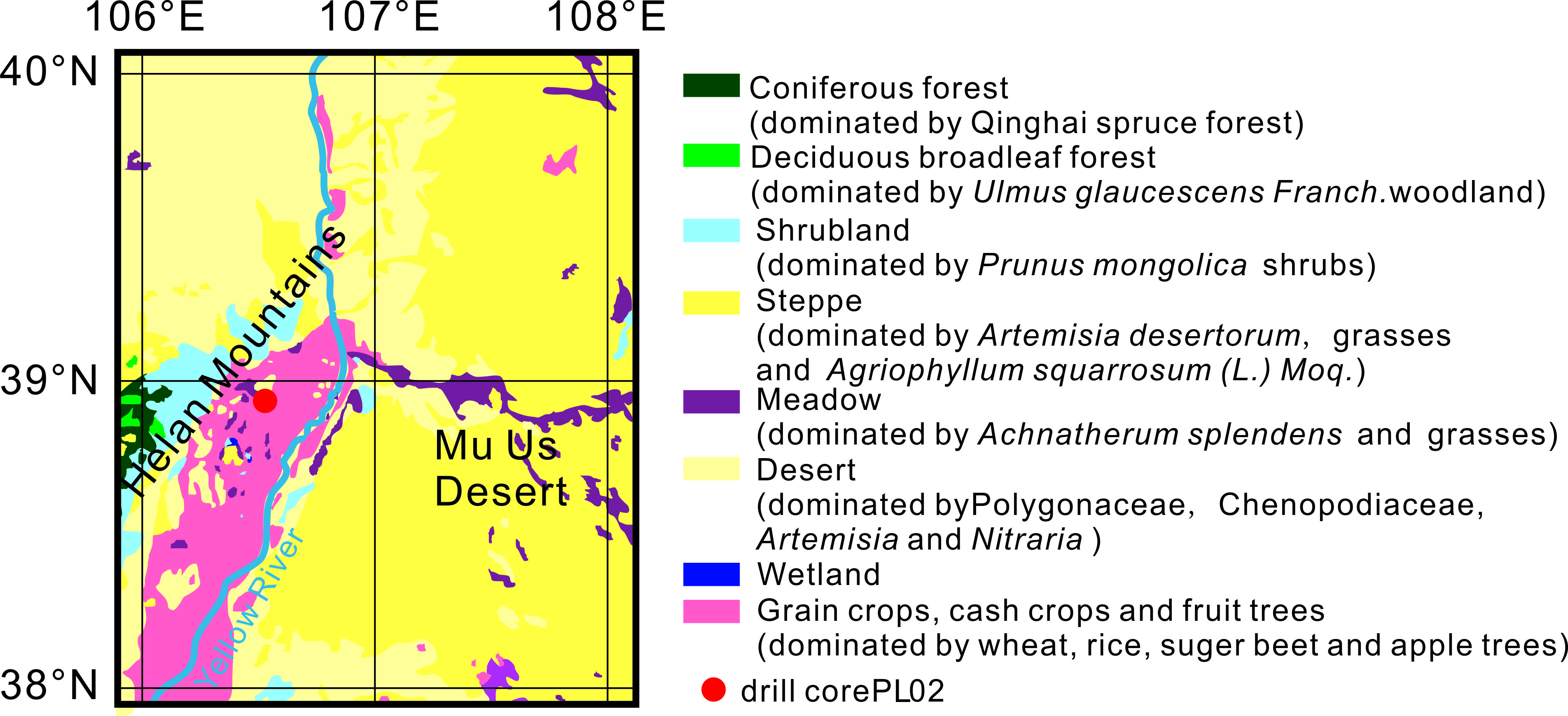
**

**Figure S2. Mean monthly temperature and precipitation in Shizuishan City for AD 1981-2010.** The temperature and precipitation data were provided by the China Meteorological Data Service Center (CMDC) (<http://data.cma.cn/>) and the figure was produced using SigmaPlot 12.0 ([http://www.sigmaplot.com](http://www.sigmaplot.com/)),

**
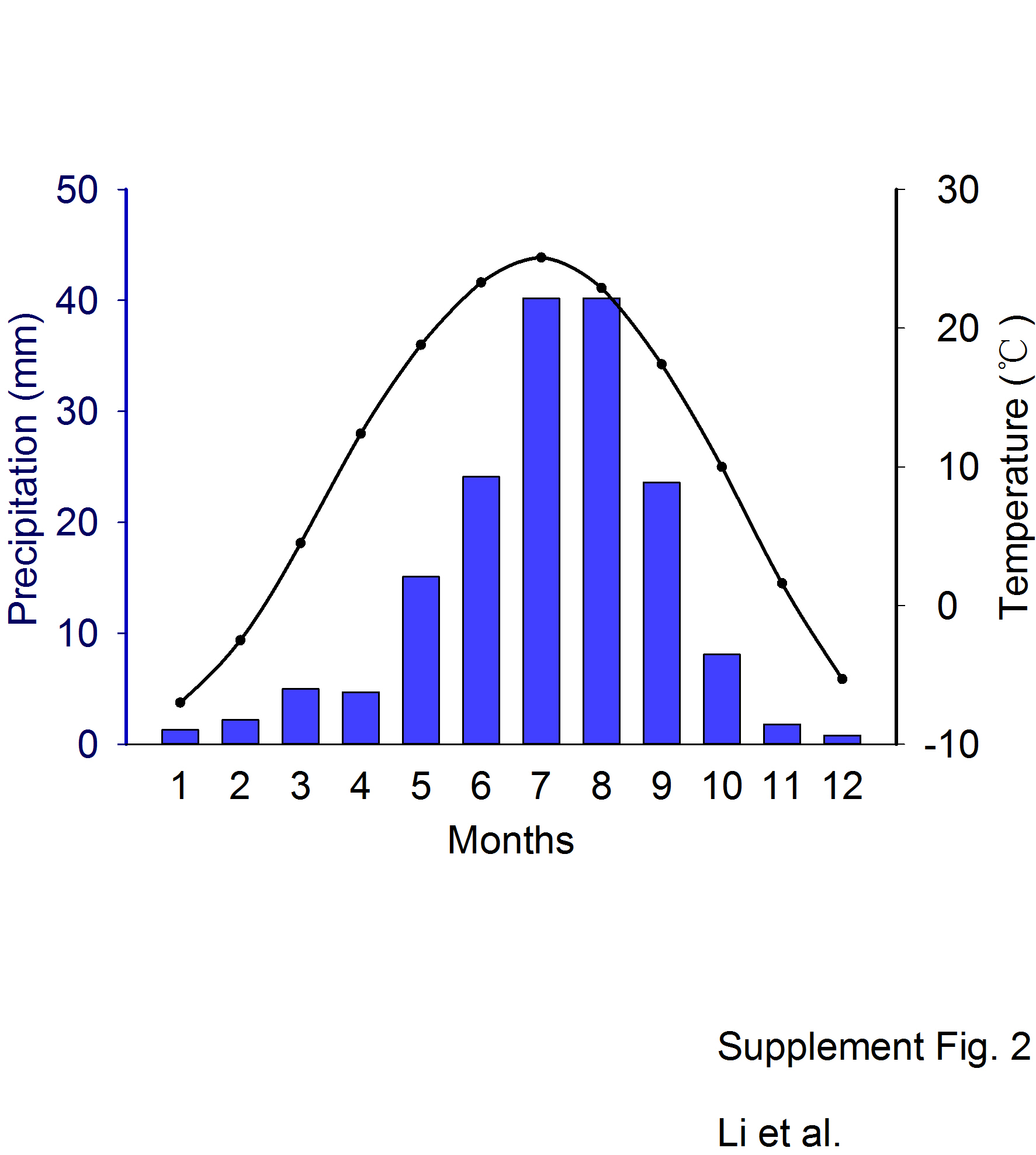
**

**Figure S3. Correlation of records from core PL02 and the LR04 δ^18^O stack (from 1.2 Ma). a**, Benthic δ^18^O stack LR04^3^; **b**, *ln* A/C; **c**, percentages of *Picea* + *Abies*. The solid line (yellow) is the *ln* A/C record based on the paleomagnetic chronology. The dashed line is the *ln* A/C based on the timescale using all tie points. The green line is the percentage of *Picea* + *Abies* based on the timescale using all the tie points. The figure was generated using SigmaPlot 12.0 ([http://www.sigmaplot.com](http://www.sigmaplot.com/)).

**
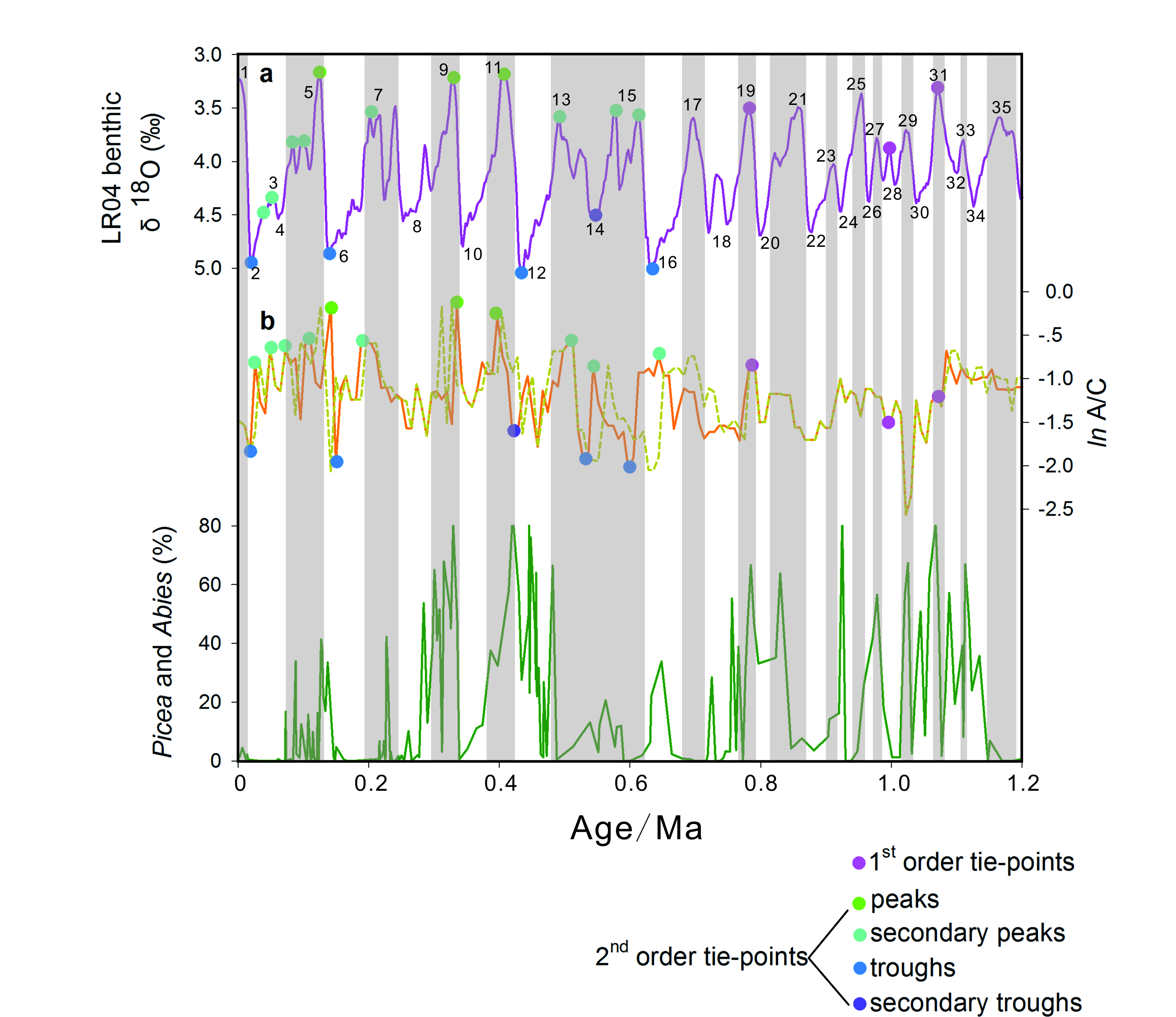
**

**Figure S4. Correlation of the *ln* A/C record of core PL02 in the Yinchuan Basin (a) and the magnetic susceptibility record of the Lingtai section in the Loess Plateau (b) (from 2.6 to 1.2 Ma).** The solid line (yellow) is the *ln* A/C record based on the paleomagnetic chronology, and the dashed line is the *ln* A/C record based on the timescale using all the tie points.

**
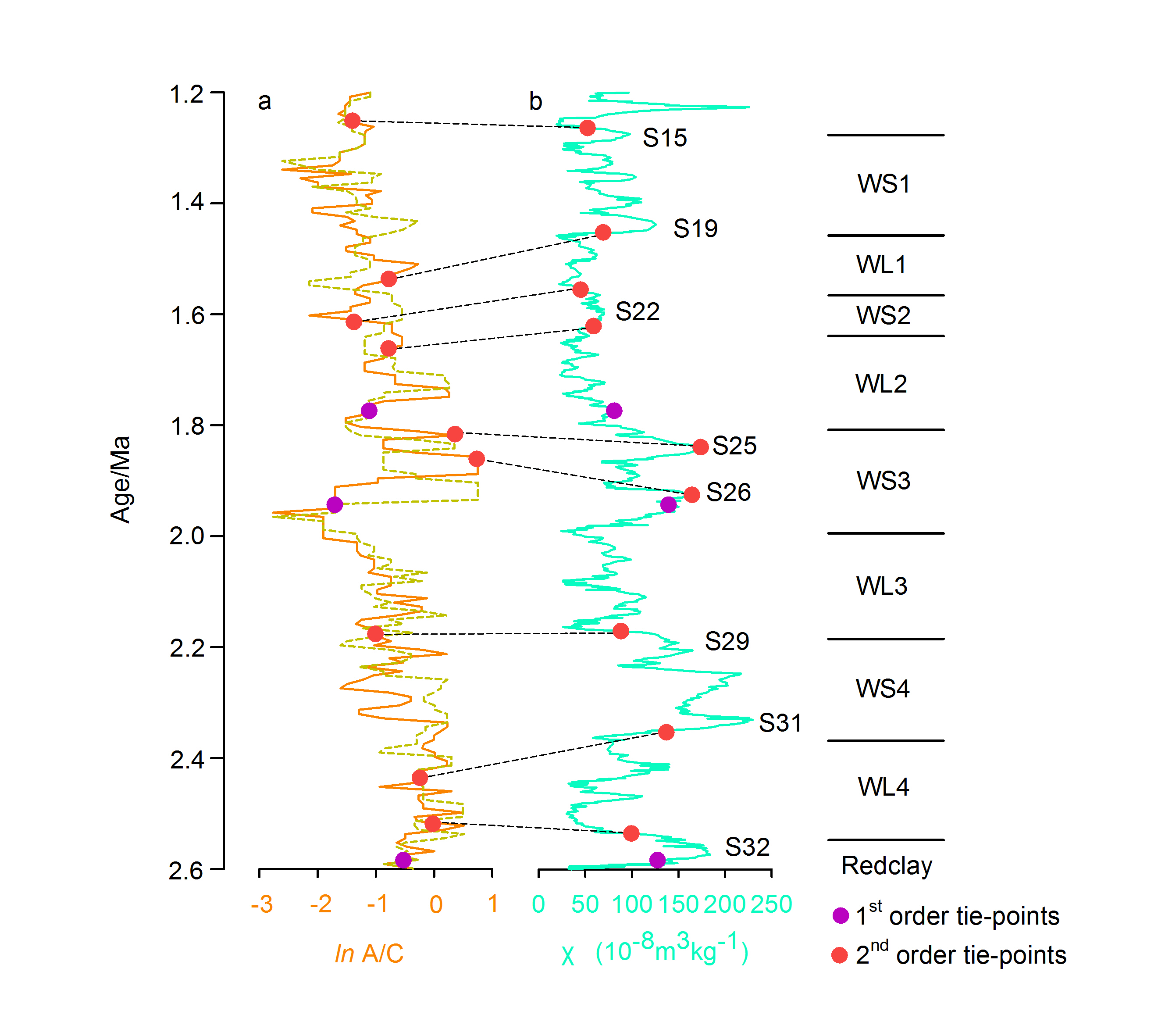
**

**Figure S5. Results of principal components analysis of the pollen data from core PL02.**

**
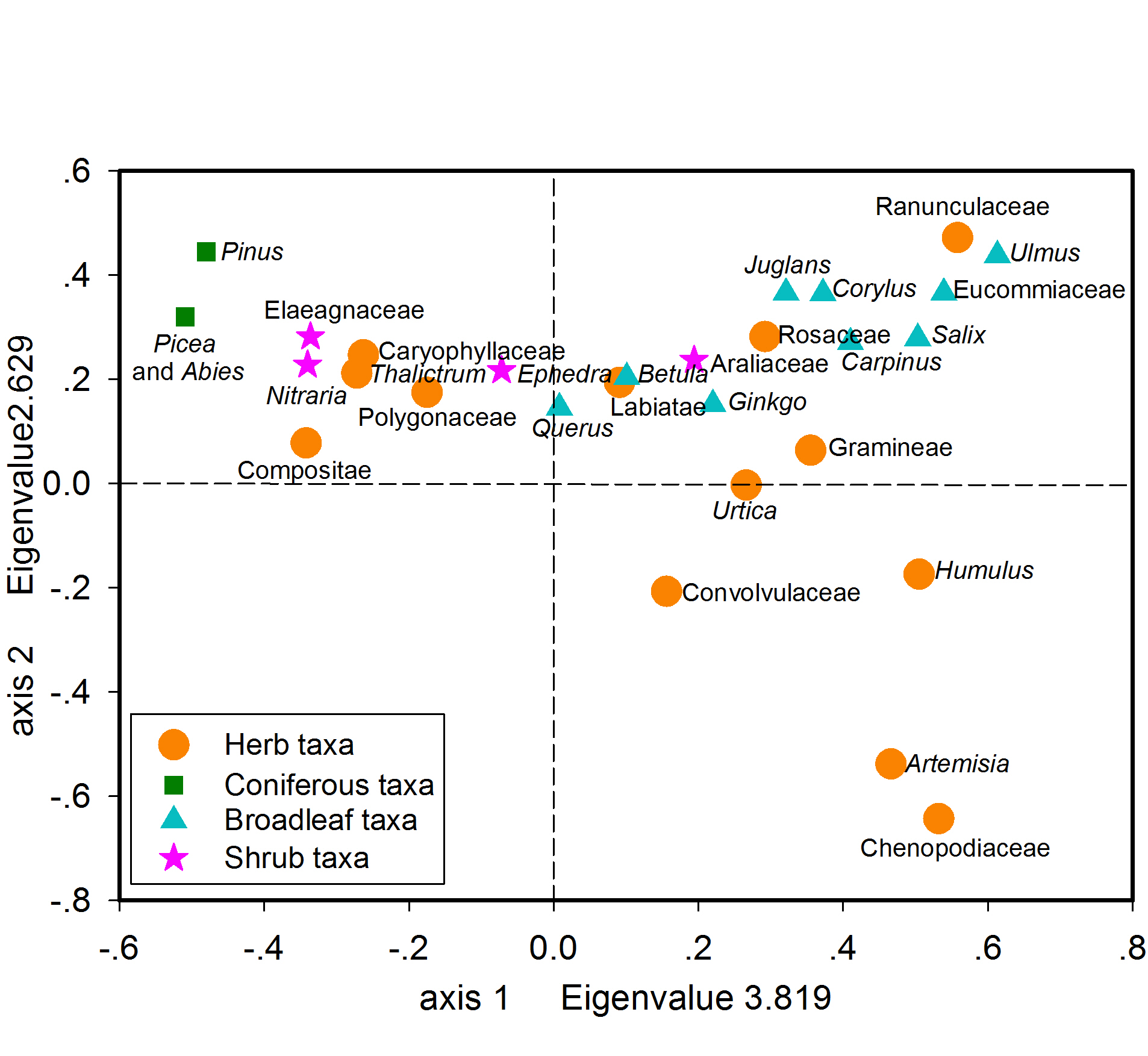
**

**Figure S6. Moving averages of the records of % *Picea* and *Abies* in core PL02. a – % *Picea* and *Abies*. b – 3-point moving average. c – 5-point moving average. d – 8-point moving average. e – 10-point moving average. f – 20-point moving average.** The figure was generated using SigmaPlot 12.0 ([http://www.sigmaplot.com](http://www.sigmaplot.com/)).

**
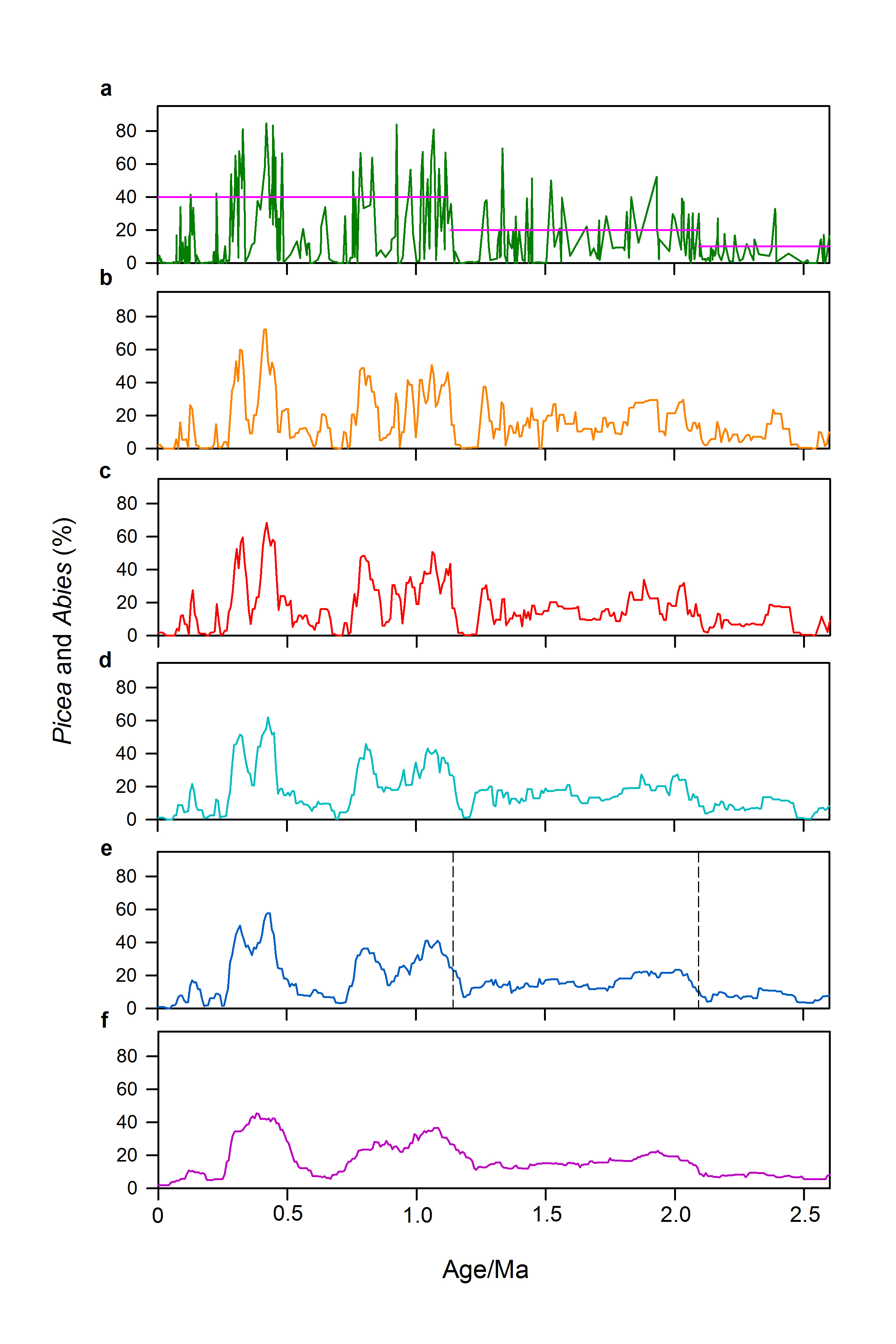
**
